# Small-World Properties in Mild Cognitive Impairment and Early Alzheimer’s Disease: A Cortical Thickness MRI Study

**DOI:** 10.1155/2013/542080

**Published:** 2013

**Authors:** Yongxia Zhou, Yvonne W. Lui

**Affiliations:** Department of Radiology, Center for Biomedical Imaging, New York University School of Medicine, 4th Floor, 660 First Avenue, New York City, NY 10016, USA

## Abstract

**Background:**

Small-world network consists of networks with local specialization and global integration. Our objective is to detect small-world properties alteration based on cortical thickness in mild cognitive impairment (MCI) including stables and converters, and early Alzheimer’s disease (AD) compared to controls.

**Methods:**

MRI scans of 13 controls, 10 MCI, and 10 with early AD were retrospectively analyzed; 11 MCI converters, 11 MCI stables, and 10 controls from the ADNI website were also included.

**Results:**

There were significantly decreased local efficiencies in patients with MCI and AD compared to controls; and MCI patients showed increased global efficiency compared to AD and controls. The MCI converters experience the worst local efficiency during the converting period to AD; the stables, however, have highest local and global efficiency.

**Conclusions:**

The abnormal cortical thickness-based small-world properties in MCI and AD as well as the distinct patterns between two MCI subtypes suggest that small-world network analysis has the potential to better differentiate different stages of early dementia.

## 1. Introduction

Brain network is an important concept in the study of brain mechanisms underlying disrupted anatomical and functional brain connectivity in many neurological diseases. It describes the human brain as a large, interacting, and complex network characterized by highly coordinated, efficient, and integrated neuronal activities among specialized and widely distributed cerebral cortices [1]. Brain small-world network is a relatively new concept, characterized by a class of regional hubs with short communication length and high clustering coefficient based on quantitative imaging values such as fMRI or EEG/MEG time series, diffusion indices, and volumetric and cortical thickness measurements [2–5]. Based on graph theory, the topological properties of small-world network include many short-distance neighboring connections and some long-distance connections that are composed of numerous nodes (vertices) related to one another by edges (connections) [6]. A few advantages of brain small-world topologies have been proposed and described [1, 7]. These include maximizing the complexity and plasticity of function while minimizing cost (i.e., less neuronal utilization); optimal synchronization of neural activity among different brain regions via central hubs; and most importantly, protecting the brain from random failure by redundant densely neighbored connections and targeted attacks under disease conditions with high resilience by high centrality and clustering. These brain neuronal networks are well balanced as well as highly-efficient with local specialization and global integration [8]. Compared with traditional decomposition method, small-world network analysis can provide globally large-scale and locally clusterwise interregional modulation information of real-time brain anatomical and functional organizations. Small-world properties such as local and global efficiency have successfully captured different neurological disease characteristics including multiple sclerosis, schizophrenia, and Alzheimer’s disease [5, 9–11].

Cortical thickness is measured with the algorithm that tries to find the globally minimal communication distance between gray and white matter media for each local point; and thus reflect an integration of global and local 3D morphology [12]. Therefore, it could provide a better accurate index for each individual subject and an actual physical characteristic for quantitative small-world property analysis [13]. Cortical thickness has been shown to be more sensitive than volume measures with increased correlations with the functional, structural, and molecular imaging (e.g., ^11^C-PiB amyloid and ^15^O-water perfusion) findings in normal aging [14, 15], mild cognitive impairment (MCI) [16–18], and Alzheimer’s disease (AD) [14, 19, 20]. All these recent findings support the notion of using cortical thickness as a potential biomarker to differentiate MCI from AD and controls in both cortical and subcortical regions [16, 21]. Further studies pertaining to the conversion from MCI to AD (estimated to about 50% within 3 years progression [22] with over 1% incidence rate of MCI [23]) have identified certain brain atrophy locations such as medial temporal lobe, especially hippocampus and entorhinal cortex, and posterior cingulate cortex to be more predictive of disease progression. One of the hallmarks of AD is amyloid pathology within the cerebral cortex [24] accompanied by cortical thinning, structural reorganization, and recruitment of new areas for functional adaptation [25–27].

We hypothesize that the degree and topographic pattern of such cortical adaptive changes are different in MCI and AD and can be detected with small-world properties based on cortical thickness. Several studies have found disrupted small-world properties in MCI or AD patients [5, 9, 28–33]; to our knowledge, no small-world analysis studies have been performed in MCI and AD with cortical thickness maps. The aims of this study were to characterize the different small-world properties in three aging populations (normal aging, MCI, and AD) and to determine the role of small-world properties in discriminating between MCI patients who will and will not convert to AD.

## 2. Materials and Methods

### 2.1. Study Population

#### 2.1.1. University of Tennessee Medical Center, Knoxville (UTMCK) Data

All subjects were recruited from existing research cohorts [34] at the Cole Neuroscience Alzheimer’s Center at the Tennessee Medical Center at Knoxville (UTMCK), and all underwent a clinical evaluation that included a history and physical examination. We recruited 10 individuals with MCI, 10 patients with early AD, and 13 healthy aging controls. The diagnosis of MCI and AD is done according to the National Institute of Neurological and Communicative Disorders and Stroke/Alzheimer’s Disease and Related Disorders Association research criteria with global scale of clinical dementia rating (CDR < 1) [35]. Table 1 details the demographic characteristics of the groups of UTMCK data with no statistical differences of age, education, and gender found between groups (*P* > 0.05).

All subjects were scanned by a Siemens 1.5 T Avanto scanner (Siemens Medical Solutions, Erlangen, Germany) with a 12-channel phased-array receive head coil with a parallel imaging technique. Head restraint with headphones and cushions was used to immobilize the subject to prevent movement artifacts and attenuate echo noise. 3D magnetization prepared rapid acquisition gradient echo (MPRAGE) axial images were acquired to be parallel to the anterior commissure (AC) and the posterior commissure (PC) line with the following parameters: TR/TE/TI = 1160/4.19/600 msec, flip angle = 15°, and resolution = 0.49 × 0.49 × 1 mm^3^. In addition, a standard fluid attenuated inversion recovery (FLAIR) imaging, a conventional resting state fMRI, and a spinecho multidirectional diffusion weighted (MDDW) protocol were also acquired to investigate if functional and structural connectivity as well as white matter lesions change with aging (results not included).

#### 2.1.2. ADNI Data

Data used in the preparation of this article were obtained from the Alzheimer’s Disease Neuroimaging Initiative (ADNI) database (http://adni.loni.ucla.edu/). The ADNI was launched in 2003 by the National Institute on Aging (NIA), the National Institute of Biomedical Imaging and Bioengineering (NIBIB), the Food and Drug Administration (FDA), private pharmaceutical companies, and nonprofit organizations as a $60 million, 5-year public-private partnership. The primary goal of ADNI has been to test whether serial magnetic resonance imaging (MRI), positron emission tomography (PET), other biological markers, and clinical and neuropsychological assessment can be combined to measure the progression of mild cognitive impairment (MCI) and early Alzheimer’s disease (AD). Determination of sensitive and specific markers of very early AD progression is intended to aid researchers and clinicians to develop new treatments and monitor their effectiveness as well as lessen the time and cost of clinical trials.

The Principal Investigator of this initiative is Michael W. Weiner, M.D., VA Medical Center and University of California, San Francisco. ADNI is the result of efforts of many coinvestigators from a broad range of academic institutions and private corporations, and subjects have been recruited from over 50 sites across USA and Canada. The initial goal of ADNI was to recruit 800 adults, ages 55 to 90, to participate in the research, approximately 200 cognitively normal older individuals to be followed for 3 years, 400 people with MCI to be followed for 3 years, and 200 people with early AD to be followed for 2 years. For up-to-date information, see http://www.adni-info.org/.

We have selected 22 individual data with a diagnosis of MCI to particularly focus on the differences of small-world network patterns of MCI subtypes from ADNI website and to match the age range of early AD group from the previously mentioned UTMCK database. Namely, MCI converters who progressed to a diagnosis of AD and MCI stables who remain cognitively stable based on MMSE and other clinical score were studied over a period of 2~3-years followup with a time interval of 3 to 6months between each study visit. Specifically, 11 MCI stables (later MMSEs were greater than 24 and at least one MMSE meet 24 ≤ MMSE ≤ 28) and 11 MCI converters (earlier MMSEs meets 24 ≤ MMSE ≤ 28 and later MMSE < 24) were selected. For MCI converters, MRI 3D T1 image data was chosen for small-world network analysis at the closest time point before conversion to AD(MMSE < 24). For MCI stables, MRI 3D T1 image data was chosen at the latest follow-up time point in the ADNI database (all MMSE ≥ 24). In addition, 10 controls (all MMSE > 28) were selected from ADNI for comparison. The demographic information of ADNI data was listed in Table 2. The three groups were age matched, and there was no statistical significance of education and gender between groups (*P* > 0.05).

The datasets included standard T1-weighted MR images acquired sagittally using volumetric 3DMPRAGEwith 0.94 × 0.94mm in-plane spatial resolution, 1.2 mm thick (matrix size 256 × 256), and 166 sagittal slices at 3T magnetic field strength. All scans were downloaded in the DICOM format and converted to the NIFTI format. Detailed information about the MR acquisition procedures is available at the ADNI website.

### 2.2. Thickness and Connectivity Computing

We used the 3D high-resolution anatomical MPRAGE data of each subject to obtain structural small-world networks based on cortical thickness measurements using Freesurfer software (http://surfer.nmr.mgh.harvard.edu/). Cortical parcellation was performed using the fully automated Freesurfer tool, and data was ascertained from the Destrieux cortical atlas [36]. Each brain was segmented to derive the boundary between the gray and the white matters, and the outer surface of the cortex (the pial surface) to represent the local fine curvatures. Bayesian maximum *a posteriori* algorithm with high flexibility was used to incorporate the observed individual surface geometry and the atlas function [37]. The thickness vector of total of 148 brain regions of interest (74 ROIs on each brain hemisphere from Freesurfer standardized landmarks) was exported from Freesurfer to derive the Pearson correlation matrix, which was used to generate small-world graphic topology. Small-world properties such as absolute clustering coefficient (*C_p_*), absolute shortest-path length (*L_p_*), relative clustering coefficient (γ), and relative path length (λ) were then computed and analyzed with in house MATLAB (http://www.mathworks.com/) scripts and programs from matlab_bgl (http://www.stanford.edu/~dgleich/programs/matlab_bgl) in each group. These particular graph metrics were chosen because they have been used to study a variety of clinically important neurodegenerative and psychiatric disorders affecting the central nervous system [5, 30, 38].

### 2.3. Small-Worldness Analysis

A few key terminologies commonly used in graph theory [3, 39–41] and this work include (1) degree (*D*): equal to the number of connections (edges) attached to each node (ROI); (2) clustering coefficients (*C_p_*): measure the number of connections of a node with its nearest nodes (neighbors), namely, the fraction of the number of a node’s neighbors that are also neighbors of each other, reflecting how efficiently the network exchanges the information at the cluster level; (3) betweenness centrality (BC):measure the number of shortest paths between pairs of other nodes that pass through the node, reflecting how efficiently the network exchanges the information at the global level. Path lengths (*L_p_*) record the length of the shortest path between a pair of nodes. BC is high for nodes that are located on many short paths in the network and low for nodes that do not participate in many short paths and are therefore more peripheral; (4) core number (*K*): defined as the largest *k* such that the node is still contained in the *k*-core (the largest subgraph comprising nodes of degree at least *k*), reflecting the rigidity or stability of the network.

**Figure 1:**
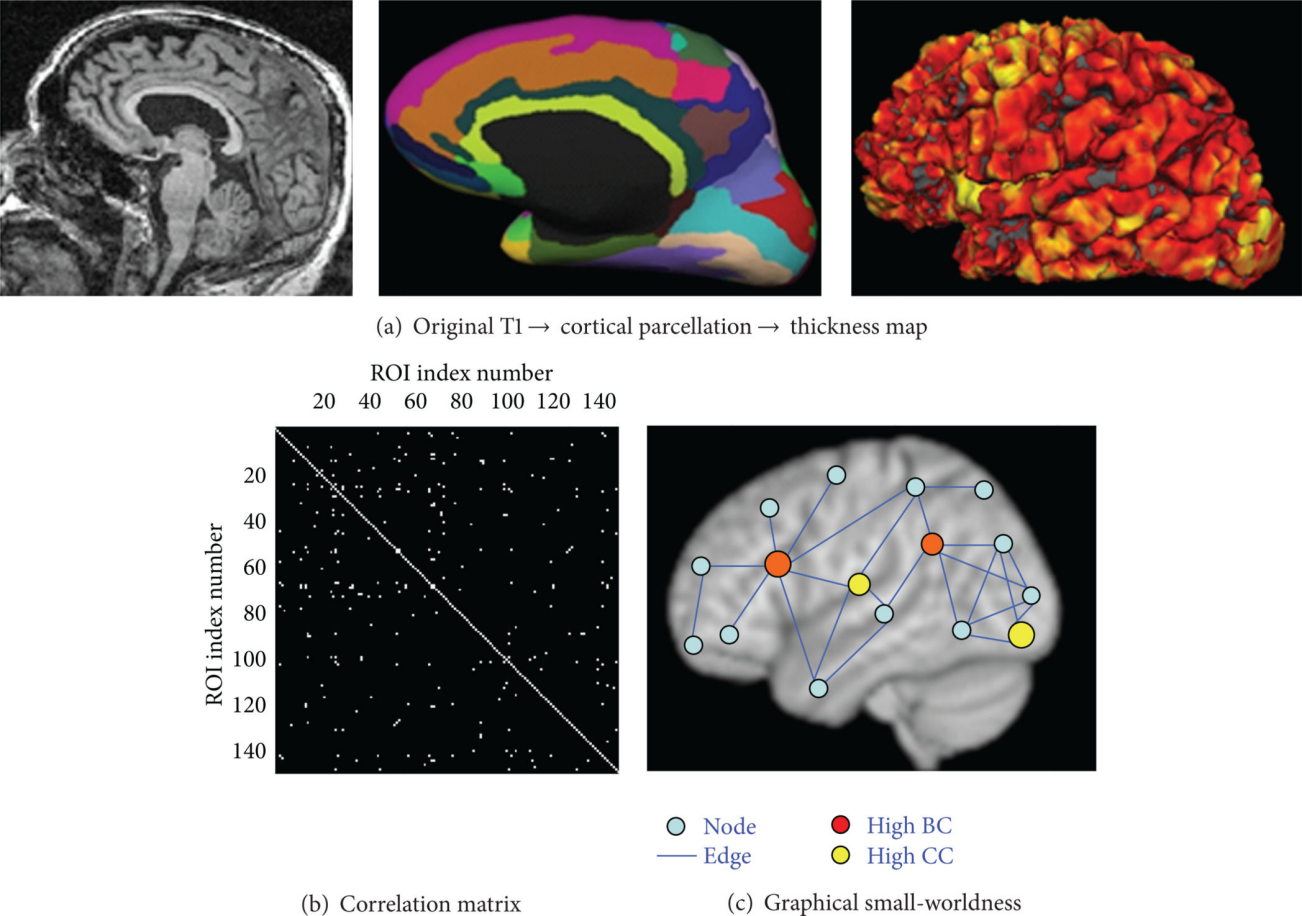
(a) The original T1 of each subject was processed with Freesurfer software to derive cortical parcellation (148 cortical ROIs) based on normalized template. The thickness map generated in Freesurfer was projected on the surface to derive the average cortical thickness of each ROI (in mm unit). (b) For each group, the thickness vectors of each ROI that consists of the thickness of all subjects within that group were derived. And the Pearson’s correlation between the thickness vectors of every two ROIs (interregional correlation) was computed and applied with a threshold with *P* < 0.05 (different *r* values based on the number of subjects in each group) to derive the correlation matrix. (c) The graphical topology of the binarized correlation matrix. Each ROI was denoted as node and each suprathreshold connectivity based on thickness vectors was denoted as edge. The nodes at the center of the graph with a “star-” shape-like edges have high betweenness centrality (BC), shown in red color, while the nodes in the center of the local cluster (satellite) have high clustering coefficient (*C_p_*), shown in yellow color.

The flowchart of the graph analysis performed in this study was sketched in Figure 1: the original high-resolution T1 of each subject was loaded in Freesurfer software to compute cortical thickness for each of 148 ROIs (Figure 1(a)). Then Pearson correlation between every two ROIs across subjects in each group was computed to derive the original correlation matrix for each group; and a threshold of *r* value from 0.2 to 0.95 was applied to this correlation matrix for all groups to investigate the small-worldness measures at different sparsity (Figure 1(b)). The binarized correlation matrix was used for small-world analysis (i.e., *L_p_, C_p_, D*, and *K*) (Figure 1(c)). A random network with the same number of degrees as the real network was generated and used as a scaling factor to compute the relative local efficiency (γ = *C_p_*/*C*_*p*-random_ ≫ 1) and global efficiency (λ = *L_p_*/*L*_*p*-random_ ≈ 1) at different sparsity levels for each group.

To compare with published findings [5], we had also repeated our small-worldness analysis using gray matter volume as a measure for UTMCK datasets.

### 2.4. Statistical Analysis

Voxel-wise 2-sample *t*-test was applied to test the mean differences of the cortical thickness among control, MCI, and AD groups. The significance level *P* was obtained with and without Bonferroni multiple-group comparison adjustment with Bonferroni correction factor of 3.

Nonparametric 2-sample rank-sum test was applied to test the median differences of the estimated small-world parameter Ϭ = γ/λ from each group after multiple-group Bonferroni correction in Matlab.

## 3. Results

### 3.1. Cortical Thickness Analysis

Based on UTMCK data, the cortical thinning ROIs in individuals with both MCI and AD compared to normal controls were found in the left posterior cingulate, left pars orbitalis, left medial occipitotemporal and lingual sulci, right middle occipital, and right lateral occipito-temporal-fusiform gyri (*P* < 0.01, Table 3).

**Figure 2:**
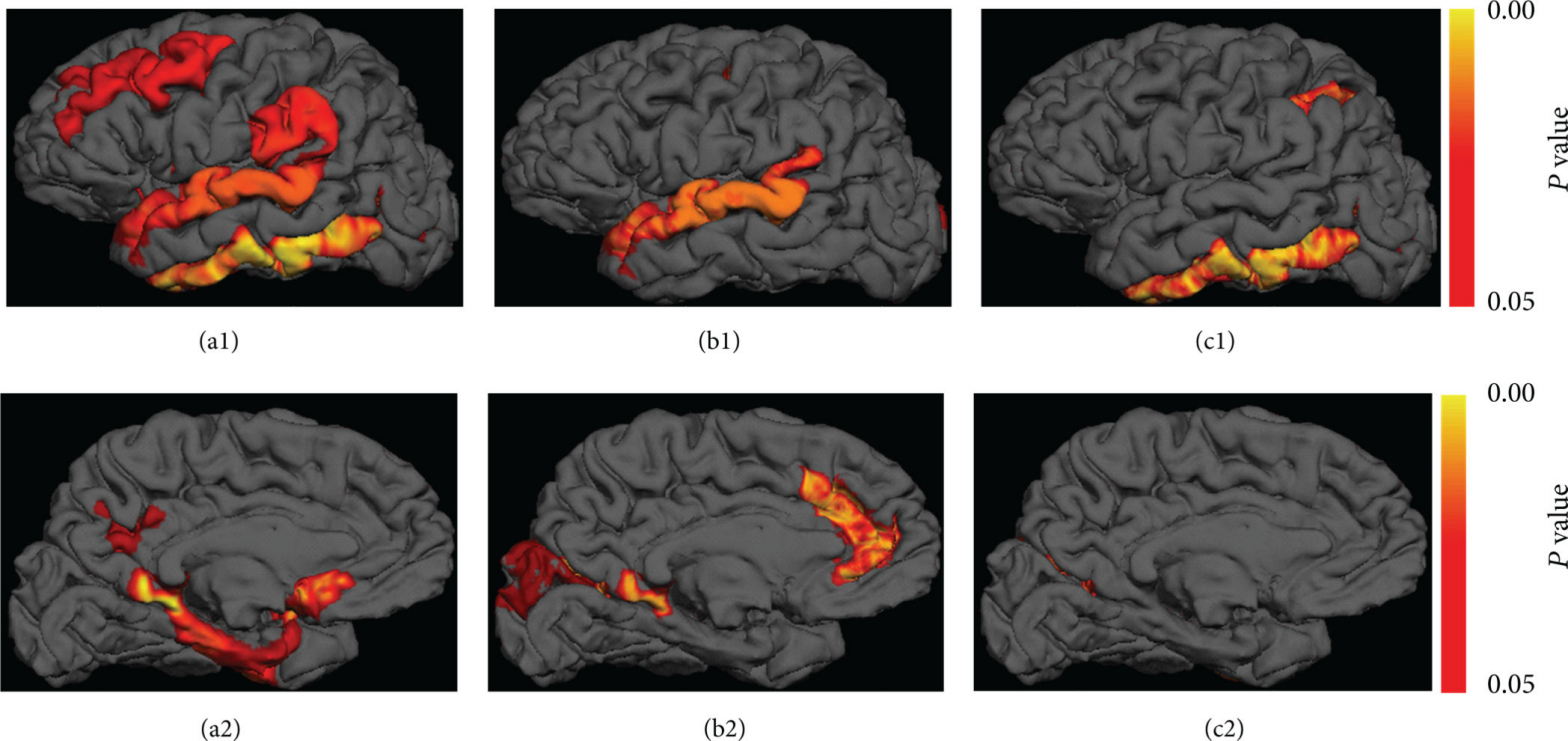
The color showed significant cortical thinning areas in MCI patients compared to controls (corrected *P* < 0.05) based on ADNI data in the left hemisphere with smoothing interpolation in Freesurfer. MCI converters less than controls in lateral (a1) and medial views (a2), MCI stables less than controls (b1, b2), and MCI converters less than MCI stables (c1, c2). The cortical thinning areas in MCI converters compared to stables include mainly the inferior temporal and pericallosal regions.

Based on ADNI data, compared to controls, there was a gradual decrease of cortical thickness from MCI stables to MCI converters (Figures 2(a) and 2(b)), and the most significant thinning areas are in the posterior cingulate and inferior temporal cortex with *P* ≤ 0.0001 comparing MCI converters to controls (results of right hemisphere not shown). The significant cortical thinning areas (*P* < 0.05) in MCI converters compared to MCI stables were found in the regions of inferior temporal gyri, middle frontal, posterior cingulate, pericallosal, left lateral occipito-temporal fusiform, and posterior collateral transverse temporal sulci, with the most significant reduction in the inferior temporal cortex with *P* < 0.01. The statistical comparison results of two MCI subtypes in comparison with control groups at significance level of *P* < 0.01 are listed in Table 4.

### 3.2. Small-Worldness Analysis

For UTMCK data, small-worldness systematic analysis showed that in controls, the precuneus and subparietal (nodes of default mode network) regions have the highest centrality and the central insular (nodes of sensory and emotional network) and posterior cingulate (nodes of default mode network) have the highest clustering coefficient, while the postcentral (nodes of somatosensory network) and superior occipital (visual network) have the highest core numbers. Other hub regions identified via BC with the criteria (≥Mean + 2 SD) include inferior triangular, superior, marginal, and orbital frontal areas; superior parietal sulcus, superior precentral, and inferior occipital regions.

**Figure 3:**
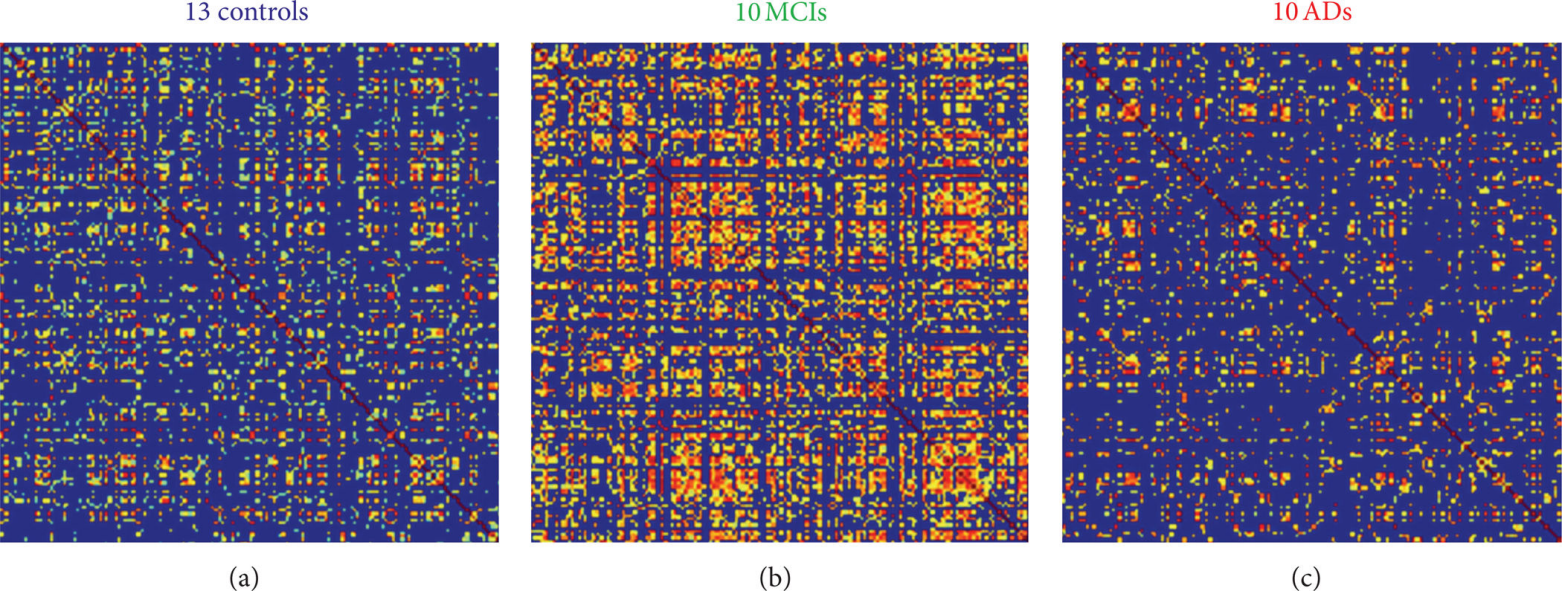
Correlation matrix and quantitative comparison of the 3 groups. Top row (a)–(c): cortical thickness-based interregional binarized correlation matrix represents human brain structural network constructed using cortical thickness of 148 ROIs within each group. The correlation matrix showed reduced connectivity in AD patients (c) compared to controls (a) and MCI patients (b); more blocked hubs in patients with MCI compared to control and AD patients (c).

**Figure 4:**
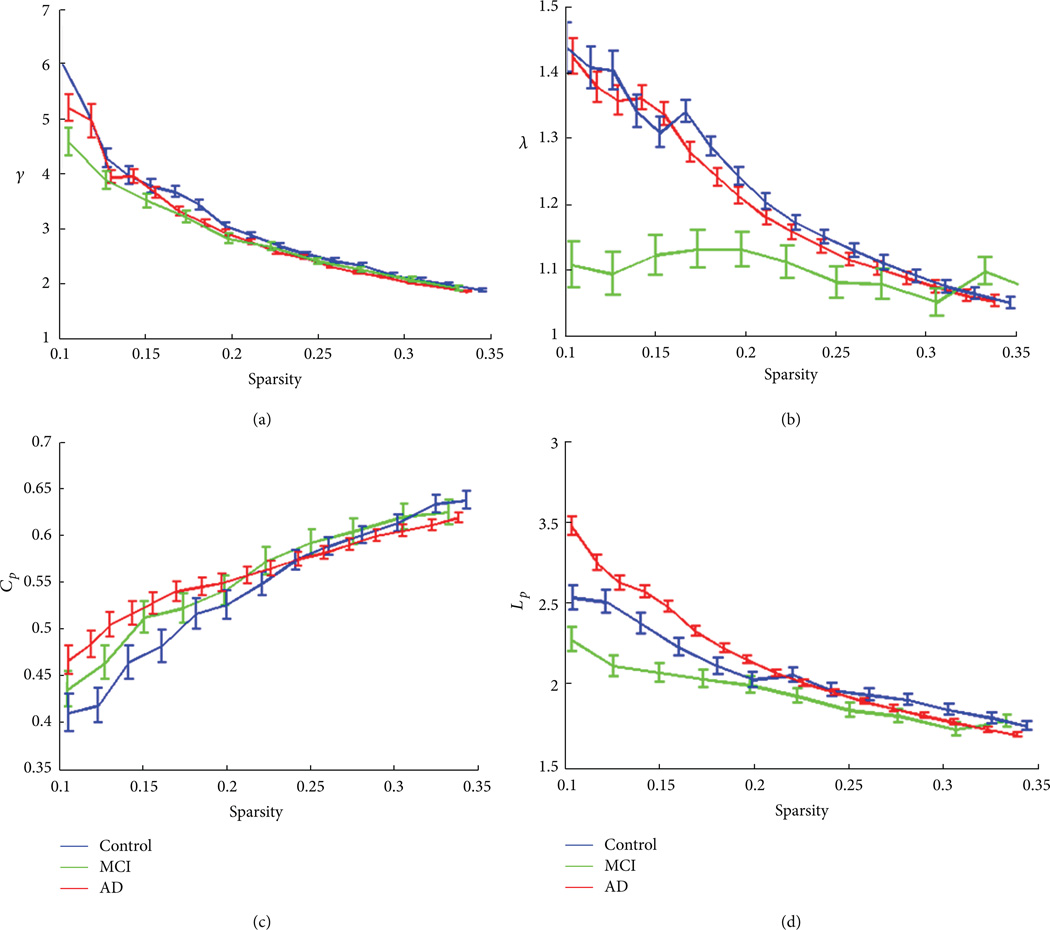
Controls (blue), MCI (green), and early AD patients (red) all showed a small-world characteristics in three population with reduction of γ (≫1) in (a) and reduction of λ (≈1) in (b) with the reduction of sparsity. AD patients showed lowest absolute clustering coefficient (a), indicating a reduction of local efficiency in AD patients. MCI patients showed lower relative cluster coefficient compared to controls (a), shortest relative path lengths (b), and lowest absolute path lengths (d) compared to AD and controls, indicating a compensatory mechanism with global efficiency in MCI stables. Error bar denotes standard deviation.

The alterations of small-world properties in patients with MCI and AD were shown in Figure 3. Compared to controls (Figure 3(a)), the correlation matrix showed more blocked structures in MCI (Figure 3(b)) and reduced interregional connectivity in AD (Figure 3(c)). The small-world decomposition analysis showed a small-worldness characteristics (γ ≫ 1, λ ≈ 1) in all three populations and also the reduction of γ and λ with the reduction of sparsity (Figures 4(a) and 4(b)). The AD patients showed lower relative and absolute clustering coefficient (Figures 4(a) and 4(c)) compared to control, indicating a reduction of local efficiency in AD patients. The MCI patients also showed lower relative clustering coefficient compared to controls (Figure 4(a)), but, on the other hand, with shortest relative and absolute path lengths compared to control and AD groups (Figures 4(b) and 4(d)). Compared to cortical thickness as a variable, small-worldness analysis using gray matter volume showed no such changes between controls and patients.

**Figure 5:**
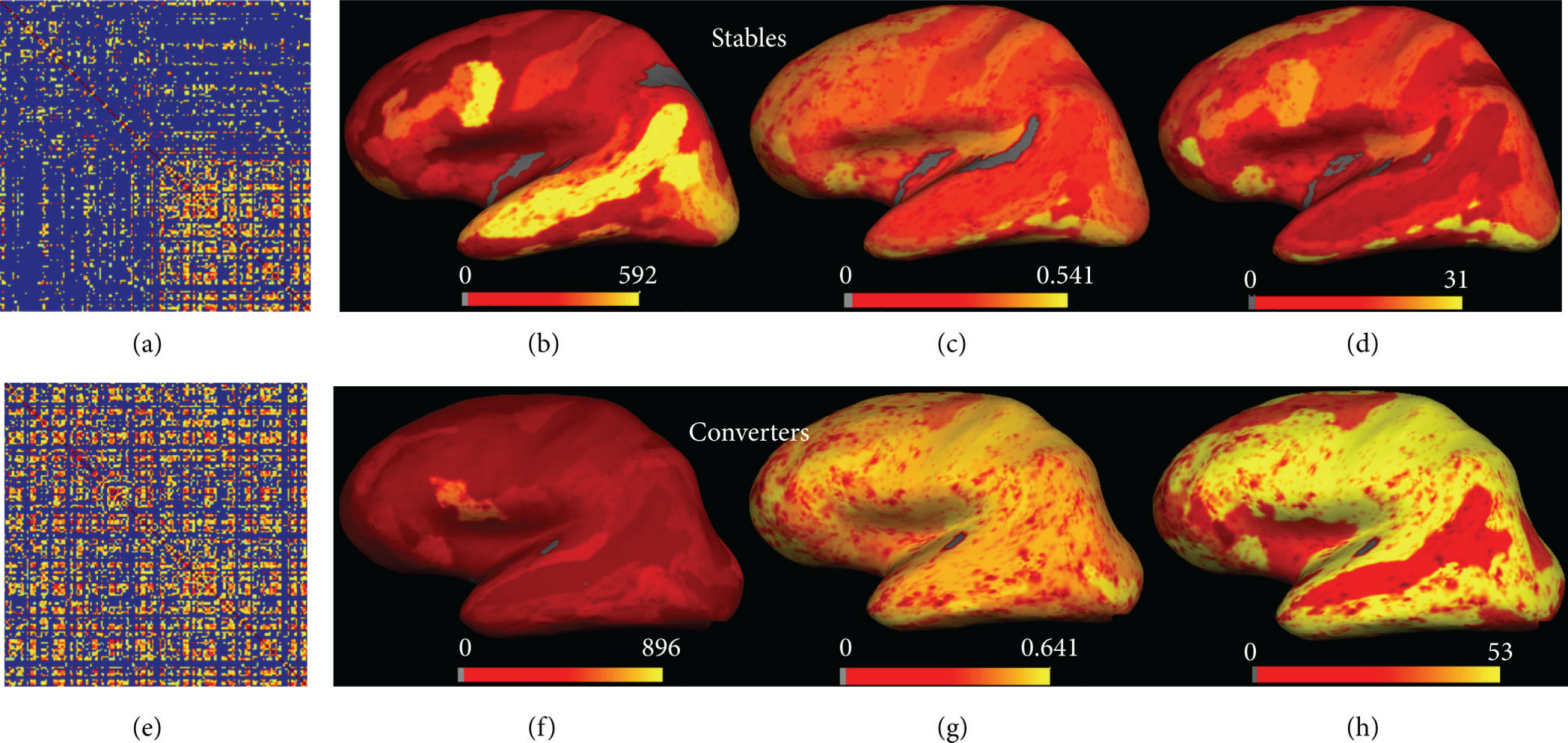
The two MCI subtypes (stables and converters) have very different small-world properties as shown by the correlation matrix (a) and (e), surface distributions of betweenness centrality (b) and (f), clustering coefficient (c) and (g), and core numbers (d) and (h).

**Figure 6:**
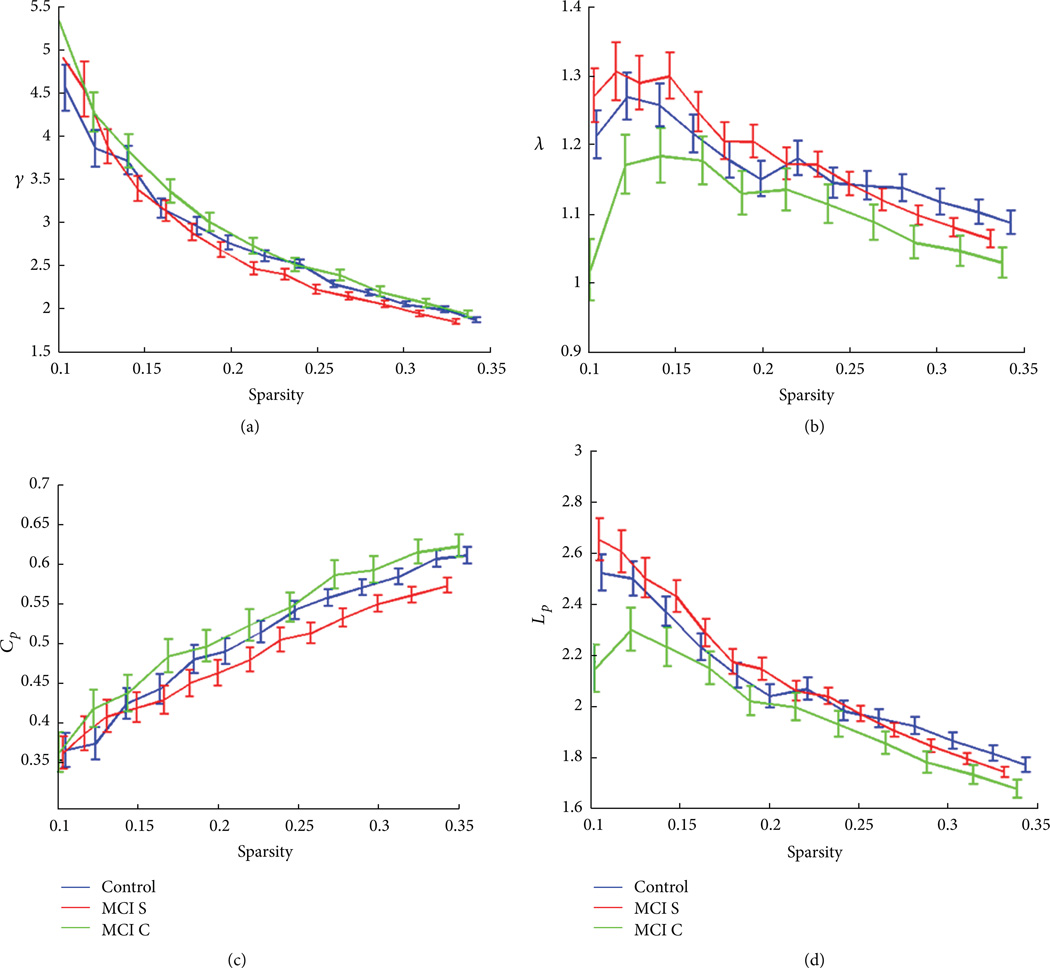
Controls (blue), MCI stables (MCI S, green), and MCI converters (MCI C, red) all showed small-world characteristics with reduction of γ (≫1) in (a) and reduction of λ (≈1) in (b) with the reduction of sparsity. MCI converters showed lowest relative clustering coefficient (a) as well as lowest absolute clustering coefficient (c), indicating a reduction of local efficiency in MCI converters. MCI stables, however, had highest relative and absolute clustering coefficient (a) and (c) as well as shortest relative and absolute path lengths (b) and (d), indicating a compensatory mechanism of local and global efficiency in these MCI stables. Error bar denotes standard deviation.

Based on ADNI data, Figure 5 showed the small-world property differences between MCI stables and MCI converters. There was a different pattern of correlation matrix between the two groups. In the MCI stables, BC and core number surface mapping (Figures 5(a)–5(d)) showed similar pattern to controls, with frontal, temporal, surrounding cingulate, and small parietal regions being the “core hubs.” In MCI converters, surface mapping (Figures 5(e)–5(h)) showed a more chaotic distribution of centrality and core number and 6(b) showed a reduction of γ (≫1) and λ (≈1) with the reduction of sparsity in ADNI data. Compared to control and MCI stables groups, MCI converters showed lowest absolute clustering coefficient (Figure 6(c)). MCI stables, however, showed highest relative and absolute clustering coefficient among three groups, as well as shortest relative and absolute path lengths (Figures 6(a)–6(d)). In addition, after correcting for multiple-group comparison, the average Ϭ parameter was significantly different between the MCI converters and stables (*P* = 0.045), indicating different brain networks recruited in two MCI subgroups.

## 4. Discussion and Conclusions

Consistent with the previously published literature [13, 14], we found reduced cortical thickness in MCI and AD compared to controls in several regions of temporal lobe. We have found a typical distribution pattern of small-world network and its key hubs in healthy control group [40]. Patients with both MCI and AD showed disrupted small-world properties with significantly reduced local efficiency compared to controls. We found no such reduction of small-worldness based on gray matter volume measure, indicating that cortical thickness is a sensitive measure to reflect the different degrees of cortical topographic changes as disease progresses from MCI to AD stage. Moreover, the finding of increased global efficiency in patients with MCI compared with controls and AD may reflect a compensative mechanism for decreased local efficiency. Such network-based findings beyond the original structural cortical thickness results might also indicate an intermediate or unpredictable status in our mixed UTMCK MCI population.

Specifically, the progressive development of MCI is a transitional state between age-related normal cognitive decline and dementia (AD) [42, 43], and we found reduced cortical thickness in converters compared to stables in a few regions involving most prominently inferior temporal and posterior cingulate. During the converting period to AD, the converters experience the worst local efficiency compared to control and stables. The stables, however, are in a more appropriate adaptive state with highest local and global efficiency compared to control and converters. These brain plasticity changes in MCI stables suggest a compensatory mechanism for the reduced cortical thickness (e.g., inferior temporal and anterior cingulated regions) found in MCI stables compared to controls. Our findings of MCI converters are consistent with the observations of He et al. and Risacher et al. [44, 45] who found that these patients were extremely vulnerable to targeted attacks on the pivotal nodes and links of the structural brain network compared to the controls.

The underlying pathological mechanisms of variable cortical thinning and the corresponding changes of its network properties are still unclear. For example, the regions in which amyloid deposition tends to be prominent from PET imaging data such as the prefrontal, gyrus rectus, precuneus, and lateral temporal regions are not consistent with the areas in which prominent thinning (e.g., temporal, fusiform, and posterior cingulate regions from our results and others) is found [38]. The regional cortical thinning may reflect pathological burdens to a more global degree of disruption of anatomic configuration of cortical mantle [38]. The small-world property alterations in MCI and AD patients revealed through cortical thickness measure are thus possibly due to the accumulation effects of amyloid or tauopathy that might occur in the early course of the disease.

Despite the present findings, our results of global brain network changes in MCI and AD patients were still preliminary given the relatively small sample size of two data cohorts. While there were no statistical age and gender differences between groups in our data cohorts, our results could improve by including more demographically similar subjects. The age and gender as well as age by gender interaction differences in cortical thickness (e.g., temporoparietal and frontal regions) and small-world network analysis (i.e., asymmetry) had been reported recently [8, 46].

Future longitudinal comparative analysis of characterizing small-world properties with different connectivity measures is needed to further elucidate the functional neuroplasticity mechanisms in MCI and AD. Extending the thickness analysis to some subcortical regions (e.g., thalamic and striatal ROIs) and testing our hypothesis in larger groups of subjects warrant further work.

The brain supports both specialized and integrative functions. Small-world network consists of well-balanced networks with local specialization and global integration. By investigating small-world property alterations, we can identify early neuroplastic changes in MCI that are not seen using the traditional decomposition methods. Therefore, this may play a role in differentiating different subtypes of MCI from AD and normal aging controls. Recently, an fMRI acquisition as short as 2 minutes can predict the small-world properties [47] and provides a new and promising tool for the application of graph theory in easy clinical set. We await more MCI subtype samples and longitudinal studies [48] to validate our findings.

## Figures and Tables

**Table 1:** Demographics of UTMCK data with means and standard deviations of age, education, and MMSE in each group. There were no statistical differences of age, education, and gender between the three groups with *P* > 0.05.

Group	AD	MCI	Control
Number of subjects	11	10	13
Age (y)	73.91 ± 11.00	68.89 ± 9.61	65.92 ± 12.11
Education (y)	12.67 ± 1.63	16.00 ± 4.00	16.14 ± 2.97
MMSE (/30)	21.36 ± 2.69	28.00 ± 1.00	29.92 ± 0.28
Male, %	36	60	23
Female, %	64	40	77

**Table 2:** Demographics of ADNI data with means and standard deviations of age, global CDR, and MMSE in each group. There were no statistical differences of age and gender between the three groups with *P* > 0.05. The MMSE latest score for MCI converters was chosen as the measure right after conversion and for MCI stables and controls was selected as the latest measure in the database.

Group	MCI converters	MCI stables	Control
Number of subjects	11	11	10
Age (y)	75.38 ± 8.65	75.43 ± 5.27	76.66 ± 9.86
Education (y)	15.46 ± 3.80	14.90 ± 3.35	16.90 ± 1.66
Global CDR	0.50 ± 0.02	0.50 ± 0.01	0.00 ± 0.01
MMSE baseline	26.09 ± 0.94	27.55 ± 1.44	29.50 ± 0.97
MMSE latest	20.09 ± 5.32	26.09 ± 0.94	29.70 ± 0.48
Male, %	73	73	40
Female, %	27	27	60

**Table 3:** Statically significant differences (*P* < 0.01) of cortical thickness (mm unit) comparing early AD, MCI, and control (CT) over 148 cortical regions with UTMCK data. And these significant differences still remain after Bonferroni multiple-group comparison adjustment with corrected *P* < 0.05.

ROI	AD	MCI	Control	*P* (AD versus CT)	*P* (MCI versus CT)
L parsorbitalis	2.31	2.36	2.56	0.007	0.003
L P cingulate	2.29	2.28	2.44	0.010	0.010
L MOTLS	1.75	1.81	2.05	0.006	0.007
R LOTFG	2.32	2.35	2.56	0.010	0.006

L: left sided, R: right sided, P: posterior, MOTLS: medial occipito-temporal and lingual sulci, LOTFG: lateral occipito-temporal fusiform gyrus.

**Table 4:** Statically significant differences of cortical thickness (mm unit) comparing MCI converters (MCI C), MCI stables (MCI S) and controls (CT) over 148 cortical regions with ADNI data. And these significance (not including italic items) still remain after Bonferroni multiple group comparison adjustment with corrected *P* < 0.05.

ROI	MCI C	MCI S	Control	*P* (MCI C versus CT)	*P* (MCI S versus CT)	*P* (MCI S versus MCI C)
L V P cingulate	2.02	2.16	2.53	* <0.001	0.011	*0.266*
L subcallosal	2.40	2.61	2.95	* 0.007	*0.067*	*0.243*
L I temporal	2.56	2.88	2.93	* 0.002	*0.561*	* 0.006
R MOTP	2.72	2.86	3.07	* 0.005	*0.112*	*0.317*
R S L temporal	2.65	2.79	2.97	* 0.006	*0.050*	*0.202*
R I temporal	2.58	2.82	3.05	* <0.001	* 0.009	0.012
R Lat OT	2.09	2.09	2.40	* 0.009	* 0.009	*0.915*
L A cingulate	2.56	2.46	2.67	*0.390*	* 0.010	*0.375*

L: left sided, R: right sided, V: ventral, P: posterior, S: superior, I: inferior, Lat: lateral, A: anterior, MOTP: medial occipito-temporal-parahippocampus, OT: occipito-temporal junction.

* Denotes significant statistical difference with *P* < 0.01.
